# Value of modified qSOFA, glucose and lactate in predicting prognosis in children with sepsis in the PICU

**DOI:** 10.1080/07853890.2024.2337714

**Published:** 2024-04-08

**Authors:** Wanyu Jia, Xue Zhang, Ruiyang Sun, Peng Li, Daobin Wang, Xue Gu, Chunlan Song

**Affiliations:** a Department of Emergency Medicine, Henan Province Engineering Research Center of Diagnosis and Treatment of Pediatric Infection and Critical Care, Children’s Hospital Affiliated to Zhengzhou University, Henan Children’s Hospital, Zhengzhou Children’s Hospital, Zhengzhou, China; b Department of Pediatrics, Zhecheng County People’s Hospital, Shangqiu, China

**Keywords:** Sepsis, age-adjusted modified qSOFA, 28-day all-cause mortality, glucose, lactate

## Abstract

The purpose was to investigate how well age-adjusted modified quick Sequential Organ Failure Assessment (qSOFA) scores paired with blood glucose and lactate levels predict the outcomes of septicemic children in the pediatric intensive care unit (PICU). One hundred children who were diagnosed with sepsis and septic shock in the PICU of Henan Children’s Hospital were eligible, and other 20 patients in the same hospital at different times were selected as a validation set. Respiratory rate (RR), heart rate (HR), capillary refill time (CRT), and Alert, Voice, Pain, Unresponsive (AVPU) scale were included in the age-adjusted modified qSOFA scoring criteria for scoring. The primary outcome was 28-day all-cause mortality. The predictive values were evaluated by the ROC curve. In the sepsis group, 50 patients were male, and 50 patients were female. The 28-day all-cause mortality rate was 52%. Fifty-one patients with age-adjusted modified qSOFA scores >1. The serum lactate level was 2.4 mmol/L, and the blood glucose level was 9.3 mmol/L. The AUCs for the age-adjusted modified qSOFA score, serum lactate and blood glucose levels for the prediction of 28-day all-cause mortality in children with sepsis were 0.719, 0.719 and 0.737, respectively. The cut-off values were one point, 3.8 mmol/L and 10 mmol/L, respectively. The AUC of the age-adjusted modified qSOFA score for the validation set of was 0.925. When the three indices were combined, the AUC was 0.817, the Hosmer–Lemeshow goodness-of-fit test showed *χ*^2^ = 2.428 and *p* = .965. When children with sepsis are admitted to the ICU, we recommend performing rapid scoring and rapid bedside lactate and glucose testing to determine the early prognosis.

## Introduction

Sepsis is a serious fatal organ dysfunction brought on by a dysregulated host response to an infection. Sepsis and septic shock are currently significant healthcare issues worldwide [1]. Millions of individuals worldwide experience sepsis and septic shock each year, and between one in three and one in six affected individuals die as a result [1]. The incidence and mortality of sepsis peak in extreme age groups, with newborns, young children and elderly individuals at the highest risk. Despite advancements in care, neonatal and pediatric mortality from severe sepsis is still more than 11% in high-income nations [2]. Globally, there are 1.2 million cases of childhood sepsis each year, 22 incidences of childhood sepsis per 100,000 person-years and 2202 cases of neonatal sepsis per 100,000 live births [2]. In high-income nations, sepsis affects more than 4% of all hospitalized patients younger than 18 and 8% of patients admitted to pediatric intensive care units (PICUs). Depending on the severity of the condition, risk factors, and geographic region, the mortality rate for children with sepsis can range from 4% to 50% [3]. Early detection, resuscitation, and care are thus important for improving outcomes for children with sepsis.

In 2016, the *American Society of Critical Care Medicine (SCCM)* and the *European Society of Intensive Care Medicine (ESICM)* jointly published the definition and diagnostic criteria for sepsis 3.0 [4]. Sepsis is a potentially fatal organ failure caused by dysregulated host responses to infection. The Sequential Organ Failure Assessment (SOFA) score is used to assess the degree of organ dysfunction. Due to the large number of scoring items included in the SOFA score, which is not conducive to rapid clinical assessment, a quick Sequential Organ Failure Assessment (qSOFA) score has also been proposed, which has been shown to have good predictive value for adult sepsis mortality [5,6]. In recent years, the qSOFA has been widely used in adult emergency departments and intensive care units to predict the incidence and prognosis of sepsis in adults [7,8]. Unfortunately, sepsis-3 has not developed qSOFA criteria for children, as the wide range of physiological monitoring indicators in children of different ages and the more complex compensatory mechanisms in children make the qSOFA inappropriate for pediatric applications. When Schlapbach et al. compared the age-adjusted qSOFA and SIRS criteria for predicting death and ICU stay in PICU patients with infection, they found that the qSOFA was not substantially better than the SIRS criteria [9]. Romaine et al. modified the age-adjusted qSOFA to create the Liverpool Rapid Sequential Organ Failure Assessment (LqSOFA), and the LqSOFA outperformed the qSOFA in identifying febrile children at risk for critical care admission and sepsis-related mortality [10].

The qSOFA score in adults is based on the respiratory rate (RR), systolic blood pressure, and the Glasgow Coma Scale (GCS). However, in contrast to in adults, hypotension is a late sign of pediatric septic shock. Several studies have shown that prolonged capillary refill time (CRT) and increased heart rate (HR) can be a marker of sepsis and can predict the severity of organ dysfunction and the risk of death [11,12]. In the United Kingdom, the Alert, Voice, Pain, Unresponsive (AVPU) scale is the usual assessment of neurologic status in pediatric emergency departments [13]. Blood lactate levels provide a valuable indirect marker of inadequate tissue perfusion, with a mortality rate of 32.0% in children with hypotension requiring vasopressors with lactate greater than 2 mmol/L and a mortality rate of 16.1% in children with lactate less than or equal to 2 mmol/L in the PICU study. It has also been shown that lactate levels above 4 mmol/L are consistently associated with mortality [3]. Blood glucose levels have also been linked to all-cause ICU mortality in patients with sepsis [14]. International guidelines similarly recommend glycemic management of septicemic patients to avoid hyperglycemia [15]. The typical physiological changes that occur in children influence the determination of HR and respiratory abnormalities. In 2005, the International Pediatric Sepsis Consensus Conference established age-appropriate cut-off levels for HR and breathing [16].

In this study, we used age-adjusted RR, HR, CRT, and AVPU as components of the age-adjusted modified qSOFA score to evaluate septicemic children admitted to the PICU, and measured their blood glucose and lactate levels to investigate the predictive value of the age-adjusted modified qSOFA score combined with blood glucose and lactate for the prognosis of septicemic children.

## Materials and methods

### Patients

This is a single-center, retrospective study. One hundred children aged less than 18 years diagnosed with community-acquired sepsis and septic shock on admission to the PICU of a grade 3 A children’s specialist hospital from 2021 to 2022 were eligible. Twenty patients with sepsis and septic shock in the same hospital in 2023 were selected as a validation set to verify the age-adjusted modified qSOFA score. The diagnostic criteria for sepsis and septic shock were performed according to ‘Expert Consensus on Diagnosis and Treatment of Septic Shock (Septic Shock) in Children (2015 edition)’ in China [17]. All children were treated according to the standardized procedure recommended by the above guidelines. Additionally, those with diabetes mellitus, hypertension, abnormal thyroid function, malignant tumors, pulmonary or heart disease, immune diseases, or genetic metabolic diseases were excluded. The written informed consents were obtained from the parents or guardians of all study participants.

We used the PASS 15 software for sample size calculation, and based on the pre-experiment, the predicted AUC for several indicators was about 0.7. With the same sample size in both groups, it was calculated that the sample size should be at least 82 cases, and we finally decided that the sample size should be 100 cases.

### Data collection

The demographic information and baseline patient characteristics were obtained from medical record data. The information on the patient’s HR, breathing rate, level of consciousness, and CRT at admission was gathered. The serum lactate and blood glucose levels measured from venous during the child’s hospitalization were collected. The time of blood glucose and lactic acid levels collection was determined according to the changing condition of the child, and the most abnormal data within 24 h of hospitalization were used.

### Calculation of scores

The age-adjusted modified qSOFA score was calculated using data from the patients’ RR, HR, CRT, and AVPU at the time of admission. And each component in the age-adjusted modified qSOFA could be scored as 0 or 1, making a total possible score of 0–4 (Table 1). Abnormal HR and RR were defined using the cut-off values given by the International Pediatric Sepsis Consensus Conference in 2005 [16] (Table 2).

**Table 1.** The age-adjusted modified qSOFA score.

	CRT	RR	HR	AVPU
0 point	<3 s	≤95th centile age-specific thresholds	≤95th centile age-specific thresholds	Alert
1 point	≥3 s	>95th centile age-specific thresholds	>95th centile age-specific thresholds	VPU

**Table 2.** The age-specific cut-off values of RR and HR (the 95th percentile).

Age group	Heart rate, beats/min		Respiratory rate, breath/min
	Tachycardia	Bradycardia	
0 days to 1 week	>180	<100	>50
1 week to 1 month	>180	<100	>40
1 month to 1 year	>180	<90	>34
2–5 year	>140	NA	>22
6–12 year	>130	NA	>18
13–18 year	>110	NA	>14

### Outcome definitions

The primary outcome was 28-day all-cause mortality, as determined by a review of the clinical notes. Twenty-eight-day all-cause mortality data were collected *via* telephone contact if patients were no longer hospitalized and had been discharged alive.

### Statistical analysis

The experimental results were statistically analyzed using SPSS 26.0 statistical software (SPSS Inc., Chicago, IL). The Kolmogorov–Smirnov test was used to test the normality of the experimental data. Normally distributed data are expressed as the mean and standard deviation, and *t*-tests or ANOVAs were used for comparisons between groups; non-normally distributed data were expressed as the median and interquartile range *M* (P25, P75), and the rank sum test was used for comparisons between groups. We assessed the prognostic ability of age-adjusted modified qSOFA combined with glucose and lactate using the area under the receiver operating characteristic curve (AUC). In general, an AUC of 0.5 indicates no discrimination, 0.7–0.8 indicates fair discrimination, 0.8–0.9 indicates excellent discrimination, and values greater than 0.9 indicate remarkable discrimination. The Youden index was used to calculate the cut-off values for each indicator [18].

## Results

### Patient characteristics, clinical outcomes, and scores

A total of 100 patients were included in this study. Fifty patients were male, and 50 patients were female. The age was 12 (6, 56) months. Ten of the 20 children in the validation set died. The age of the 20 children was 23.50 (7.25, 48.00) months, of which 11 were males. Sixty-three of them were respiratory tract infections, 24 had central nervous system infections, eight had gastrointestinal infections, three had skin mucosal infections, and two had bloodstream infections. Septic shock occurred in 33 out of 100 children. Fifty-two of the 100 children died within 28 days of hospitalization, representing a 52% mortality rate. Fifty-one patients had age-adjusted modified qSOFA scores ≥2. The serum lactate level was 2.4 (1.3, 7.1) mmol/L, and the blood glucose level was 9.3 (7.2, 11.8) mmol/L. The specific data of the survival group and the death group are shown in Tables 3 and 4. The age-adjusted modified qSOFA scores, lactate concentration, and blood glucose concentration were significantly lower in the survival group than in the death group, and the difference was statistically significant.

**Table 3.** The patient characteristics, clinical outcomes, and scores.

Characteristics	Total	Survival group	Death group	*p*
*N*	100	48	52	–
Age (months)	12 (6, 56)	13.5 (4.5, 61.75)	12 (6.25, 36.75)	.989
Male sex, *n* (%)	50 (50%)	29 (60.41%)	21 (40.38%)	.045
Age-adjusted modified qSOFA scores				<0.001
0, *n* (%)	23 (23%)	17 (35.42%)	7 (13.46%)	
1, *n* (%)	26 (26%)	15 (31.25%)	12 (23.08%)	
2, *n* (%)	32 (32%)	14 (29.17%)	16 (30.77%)	
3, *n* (%)	12 (12%)	1 (2.08%)	11 (21.15%)	
4, *n* (%)	7 (7%)	1 (2.08%)	6 (11.54%)	
Serum lactate (mmol/L)	2.4 (1.3,7.1)	1.55 (1.00,3.00)	4.5 (1.78, 13.98)	<.001
Blood glucose (mmol/L)	9.3 (7.2,11.8)	8.00 (6.25,9.78)	10.65 (8.25, 15.30)	<.001

**Table 4.** The age-adjusted modified qSOFA score of validation set.

Characteristics	Survival group	Death group	*p*
*N*	10	10	
Age-adjusted modified qSOFA scores			0.001
0, *n*	5	0	
1, *n*	5	3	
2, *n*	0	3	
3, *n*	0	1	
4, *n*	0	3	
Serum lactate (mmol/L)	2.05 (1.68, 2.45)	4.75 (2.65, 11.50)	.007
Blood glucose (mmol/L)	7.60 (5.57, 8.20)	11.5 (8.15, 15.42)	.002

#### ROC curve of age-adjusted modified qSOFA score, serum lactate and blood glucose

The AUC for the age-adjusted modified qSOFA score for the prediction of 28-day all-cause mortality in children with sepsis was 0.719 (0.620, 0.804). The Hosmer–Lemeshow goodness-of-fit test showed *χ*^2^ = 2.423 and *p* = .489, suggesting that the predicted values were in general agreement with the actual values. The AUC of the age-adjusted modified qSOFA score for the validation set of 20 children was 0.925, further demonstrating the good predictive value of the score for 28-day all-cause mortality in children with sepsis.

**Figure 1.**
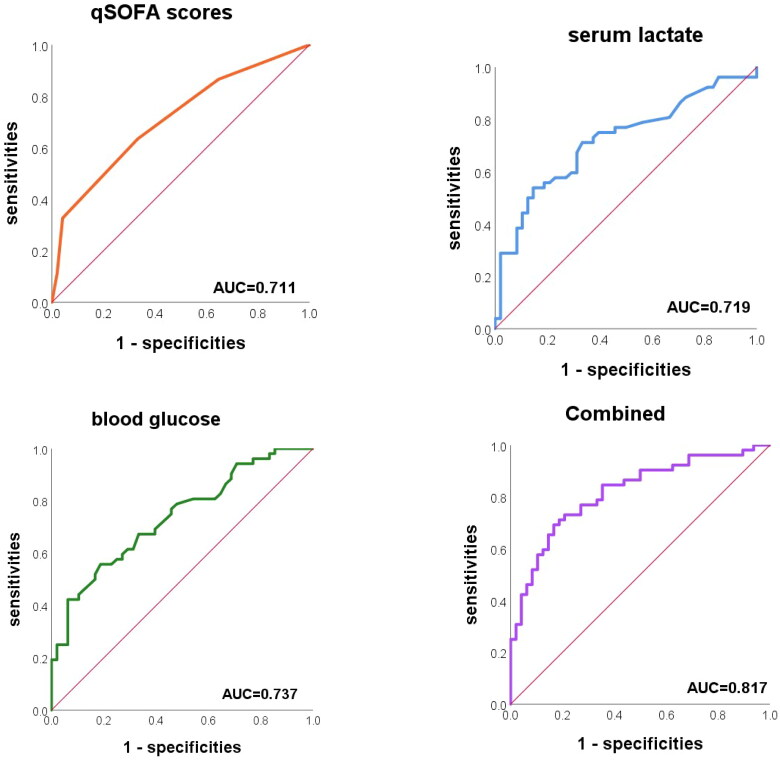
ROC curve of age-adjusted modified qSOFA score, serum lactate and blood glucose.

When serum lactate and blood glucose were utilized individually for the diagnosis and prediction of 28-day all-cause mortality, the AUCs were 0.719 (0.618, 0.820) and 0.737 (0.641, 0.833), respectively. When the three indexes were combined, the AUC was 0.817 (0.727, 0.888), which was significantly higher than that of the single detection (combined-age-adjusted modified qSOFA score *p* = .012, combined-serum lactate *p* = .034, and combined-blood glucose *p* = .040) (Figure 1). The Hosmer–Lemeshow goodness-of-fit test showed *χ*^2^ = 2.428 and *p* = .965, indicating that the goodness of fit is good.

Through ROC curve analysis, we determined the age-adjusted modified qSOFA, lactate, and blood glucose cut-off values that were highly sensitive and specific in predicting 28-day all-cause mortality. Table 5 displays the sensitivity, specificity, cut-off values positive predictive values (PPVs) and negative predictive values (NPVs) of these indices for 28-day all-cause mortality. The cut-off values of age-adjusted modified qSOFA score, serum lactate concentration and blood glucose concentration were 1, 3.8 mmol/L and 10 mmol/L, respectively.

**Table 5.** The sensitivities, specificities and cut-off values.

	Cut-off values	Sensitivities	Specificities	Youden’s index	PPV	NPV
Age-adjusted modified qSOFA scores	>1	63.46%	66.67%	0.3013	62.75%	67.35%
Serum lactate	>3.8 mmol/L	53.85%	85.42%	0.3926	63.08%	80.00%
Blood glucose	>10 mmol/L	55.77%	81.25%	0.3702	62.90%	76.32%
Combined detection	–	69.23%	83.33%	0.5256	–	–

## Discussion

Sepsis is a common pediatric critical illness, and a public health problem shared by children worldwide [19]. The fatality rate of sepsis is substantially greater in children than in adults [2,20]. The rapid progression of childhood sepsis, high morbidity and mortality rates, accurate assessment and early identification of critically ill children, and early and timely active intervention are of great significance to improve the prognosis of childhood sepsis and reduce mortality. In this study, the 28-day all-cause mortality rate for children hospitalized in the intensive care unit with sepsis was 52%. The higher mortality rate of septicemic children in this study may be due to the fact that this study included children with sepsis with more severe symptoms in the PICU. Some of these children were admitted to hospital already in a state of septic organ damage, and 33% of them had septic shock during admission, resulting in a higher mortality rate than in other medical institutions despite aggressive and professional treatment. The results of this study showed that the age-adjusted modified qSOFA score, blood glucose level, and serum lactate level were predictive of 28-day all-cause mortality in children with sepsis, and all three indices had comparable predictive power. A modified score equal to or greater than 2, serum lactate greater than 3.8 mmol/L, and blood glucose greater than 10 mmol/L were all risk factors for 28-day all-cause mortality in sepsis patients.

Quick scoring is not appropriate for children due to physiological differences between children and adults; thus, numerous academics have developed age-adjusted scoring standards for children that have some diagnostic and prognostic predictive value [21]. There are no uniform age-adjusted scoring criteria for children. In this study, HR, RR, CRT, and AVPU were included in the scoring criteria by combining previous research results and clinical validation, and the results were found to have moderate predictive value for 28-day all-cause mortality (AUC = 0.711). Our results confirm that an age-adjusted modified qSOFA score greater than 1 can be utilized as a preliminary prognostic assessment for children with sepsis admitted to the critical care unit. In addition, the bedside parameters in the age-adjusted modified qSOFA scoring standard in this study are easy to obtain, which is very conducive to clinical application. We recommend that early and intensive treatment be given to children whose age-adjusted modified qSOFA score is >1.

The predictive values of lactate and glucose for sepsis in our study are generally consistent with previous studies. Numerous investigations have demonstrated that, in both adult and pediatric sepsis, serum lactate concentrations are related to patient survival and/or organ failure [22–24], and elevated lactate is part of the sepsis-3 definition of septic shock. In a study conducted in the PICU, the mortality rate in hypotensive children with a lactate concentration greater than 2 mmol/L and who required vasopressors was 32.0%, compared with 16.1% in children with a lactate concentration less than or equal to 2 mmol/L [25]. According to other investigations, lactate concentrations exceeding 4 mmol/L are consistently linked to mortality [26]. Hayashi et al. showed that the maximum lactate concentration threshold of 3.05 mmol/L at 24 h after ICU admission had the highest predictive value for mortality [27]. Combining previous studies and our findings, we suggest that a lactate concentration >3.8 mmol/L can be used as a predictor of 28-day mortality in patients with sepsis.

The organisms of children with sepsis are in a high catabolic state, particularly those with issues related to glucose metabolism, and the most common clinical sign is stress hyperglycemia [28]. Acute stress causes the organism to develop disorders of energy and substance metabolism, which leads to an overproduction of systemic inflammatory mediators and to metabolic abnormalities associated with hyperglycemia, also known as stress hyperglycemia. According to a growing body of evidence, blood glucose levels are connected to the severity of sepsis and are one of the independent risk factors for sepsis fatality [29,30]. Additionally, our study demonstrated that blood glucose levels in children with sepsis were related to 28-day all-cause mortality and that blood glucose levels greater than 10 mmol/L were one of the predictors of 28-day all-cause mortality.

In our study, the age-adjusted qSOFA score was easy to calculate, but this occurred at the expense of accuracy. Our study also confirmed the high predictive value (AUC = 0.817) of the age-adjusted modified qSOFA score combined with lactate and glucose for 28-day all-cause mortality in sepsis patients, with improved sensitivity and specificity. As a result, we propose that rapid scoring and rapid bedside lactate and glucose testing be performed on the admission of children with sepsis to the intensive care unit for initial prognosis prediction.

Our study also has some shortcomings: this study was retrospective and may have selection bias. A prospective multicenter study with a larger sample size of children with ICU sepsis should be conducted to further determine the use of the age-adjusted modified qSOFA score in the PICU. Meanwhile, a large cohort is needed to develop and validate a new score by combining qSOFA, lactate, and glucose, and compare it to the original pediatric SOFA score.

## Conclusions

In conclusion, our study showed that the age-adjusted modified qSOFA score, serum lactate concentration, and blood glucose concentration have a predictive value for 28-day all-cause mortality in children admitted to the PICU with sepsis, and that combined testing can improve the predictive value, sensitivity, and specificity. When children with sepsis are admitted to the intensive care unit, we recommend performing rapid scoring and rapid bedside lactate and glucose testing to determine the early prognosis.

## Data Availability

The datasets generated during and/or analyzed during the current study are available from the corresponding author upon reasonable request.
